# Miscellany

**Published:** 1892-09

**Authors:** 


### Miscellany.

### THE NEW YORK STATE LAW CONTROLLING THE PRACTICE OF MEDICINE.

We take pleasure in printing the following letter from the first number of the *American Therapist*, a new journal devoted to modern therapeutics, edited by John Aulde, M. D., published in New York, and which appeared in July, 1892. This letter is ample testimony, from a disinterested source, of the usefulness and practical good working of the law providing for separate State license to practice medicine in the State of New York:

*To the Editor*:—In the report for 1890 of the Board of Health of the State of Illinois on Medical Education in the United States and

Canada, it is shown that in the decade from 1879 to 1889 there were 128,005 matriculants in the various medical colleges, both regular and sectarian, with 40,495 graduates; the medical profession of this country being augmented by an annual addition of more than 4,000 physicians.

During the period mentioned there has been a slight increase in the number of colleges; while those that exact educational requirements for matriculation, and attendance on three or more courses or lectures, have increased almost 300 per cent. And a better indication of the determination of the colleges to send out well-equipped graduates, is shown by more than twenty per cent. of the colleges now requiring four years of medical study as a requisite for graduation.

This action by the colleges is in conformity with the increasing number of States that have passed laws regulating the practice of medicine; the advantages of such legislation being so apparent, argument in its favor is supererogatory.

After many efforts, and at a rather late date, the State of New York has enacted laws controlling the practice of medicine that seem to promise much for the future. These laws may be divided into three sections; the first providing for the preliminary education of medical students; the second regulating the licensing and registration of physicians and surgeons; and the third providing for the establishment of boards of medical examiners.

The first section requires that a matriculate must be a graduate in arts, science, or philosophy, from some college in good standing; or, in lieu thereof, he must have passed an examination in arithmetic, grammar, geography, orthography, American history, English composition, and natural philosophy, or in some substantial equivalent of one or more of the foregoing, the examination being conducted under the authority of the Regents of the University of the State of New York.

The second section requires the applicant for registration to be over twenty-one years of age; to have certificates of moral character from two legally licensed physicians; a diploma conferring the degree of M. D. from some legally incorporated medical college in the United States, or a diploma or license conferring the right to practise medicine and surgery in the country in which it was granted; evidence that the applicant has studied medicine three years, including *three* courses of lectures in different years, in some legally incorporated medical college or colleges, no two of the above courses of lectures beginning or ending in the same calendar year; the candidate must pass examinations in anatomy, physiology, hygiene, chemistry, surgery, obstetrics, pathology, and diagnosis, therapeutics, practice, and materia medica. In the last three departments, the questions must be in harmony with the tenets of the school selected by the candidate. A fee of $25 must be paid in advance.

The third section provides that boards of examiners shall represent the medical society of the State and the homeopathic and eclectic medical societies. Each board consists of seven members, each of whom serves for three years, appointed by the Regents of the University from a list of at least fourteen nominees submitted by each society.

One of the most important features of the State law is that the Regents may accept licenses from other State Boards of medical examiners maintaining an equal standard, without further examination. The general adoption of this comity would make the reformation of medical practice much more national in character; and it would seem to be the feasible extension of an existing principle, for in all States having such laws, medical officers of the army, navy, and marine hospital service are exempted from such examinations, those of the government being accepted as evidence of their professional competency.

I am indebted to the courtesy of Ralph W. Thomas, the law and medical examiner for the University, for the information that since the law has gone into effect there have been eighty-seven applicants for admission to the examination, of whom eighteen were rejected because they failed to meet the requirements of preliminary education, and of three courses of medical lectures in three different years. He states that in New York it is preferable "to require a high standard of education before admission to the medical examinations, and then a fair test of the candidate's ability to practise medicine by the examination, rather than a low and imperfect preliminary and medical standard of education and an unduly severe medical examination."

The public, as well as the medical profession, has been confronted for years by an increasing number of medical colleges, offering nearly every inducement excepting more extended and thorough teaching to the matriculants. The consequence has been that the value of the degree of Doctor of Medicine has been lessened, and, most important, the public has been subjected to not inconsiderable risks from the exercise of their vocation by poorly qualified practitioners. To recognize the existence of an evil suggests a remedy, and the State has as much right to prescribe the requirements necessary to practise medicine, as it has those to practise law, or those to pursue a vocation that may endanger the health or welfare of its citizens.

Examinations do not necessarily furnish evidence that the successful candidate can practically apply his knowledge. But the chances are greater that one possessing knowledge will successfully meet an emergency than that a partially informed person will do so.

It does not seem that the law of New York exercises any invidious distinction; nor could any of its provisions be omitted without weakening its force.

The application is satisfactory to the public and the profession, and there is every indication that it is accepted, as is sunshine or any other salutary feature of daily life.

S. T. Armstrong, M. D., Ph. D.,

*Visiting Physician Harlem Hospital; Secretary Section of Public Health, N. Y.; Academy of Medicine, Etc.*

155 West Fifty-fourth Street, New York.

---

Medicine for the Hair.—To brush and brush and still to brush is the best medicine for the hair, remembering always that it is the hair and not the scalp which is to receive this treatment, writes Mrs. Mallon in the July *Ladies' Home Journal.* Upon the brush used depends a great deal. In the first place, it must be immaculately clean, and one's brushes should be washed as religiously as is one's face. The comb should be coarse, so that it will disentangle the hair if it is snarled, but if the hair is well brushed the comb really is of very little use. A fine comb is never advised. The brush should have long, soft bristles that go through the hair, taking with them every particle of dust and leaving behind them a glow that is beautiful.

---

To Wash Silk Undergarments.—To three gallons of warm water add three tablespoonfuls of household ammonia, writes Mrs. Parloa in her valuable department, "All Around the House," in the August *Ladies' Home Journal.* Let the silk garments soak in this for twenty minutes, then rub soap on the parts which are the most badly soiled, and wash the articles with the hands. Never rub them on a board. Rinse in two waters, wring dry, and hang on the line. When nearly dry, take in and fold, and, if possible, iron within a few hours. Never let an iron come in contact with the silk. Lay a piece of cloth over the fabric, and iron on that.

---

The Medical and Surgical Register of the United States.—R. L. Polk & Co., Detroit, Mich., have now in course of compilation the third edition of this valuable publication. The Medical and Surgical Register has become a standard work, and occupies its field exclusively; it gives a complete record of the physicians of the United States, colleges and date of graduation, besides much other valuable information of interest to the profession. It is the only work that has ever made the attempt to record the physicians of the United States according to the medical college training each individual has received, and is remarkably free from the mistakes inseparably connected with the preparation of such a volume. The work is worthy the support of the profession, and we trust that every physician will respond promptly to the request of the publishers for data which will aid materially the compilation of the work.—*Medical Fortnightly.*

---

Woman's Russet Shoe.—Fashion has decreed that soft, undressed leather shoes in the natural russet shade may be worn all the day long, unless, indeed, one is gotten up very gorgeously for some special occasion, writes Mrs. Mallon in the August *Ladies' Home Journal.* I cannot recommend a white shoe, for even the foot of a Cinderella looks large and ill-shaped in it. For wear with an all-white costume, nothing is so pretty as a black patent leather shoe, fitting one well and being sufficiently large so that the foot is not forced into the narrow, pointed toe.

---

The Chicago *Medical Recorder*, edited by Dr. Archibald Church, and previously published by W. T. Keener, of Chicago, is now being published by the M. H. Kauffmann Medical Publishing Company.

